# Alkalinity–silicon ratio as an assessment factor for the efficiency of silicate slags in wetland rice

**DOI:** 10.1038/s41598-021-84922-2

**Published:** 2021-03-05

**Authors:** Thoppil Sreenivasan Sandhya, Nagabovanalli Basavarajappa Prakash

**Affiliations:** Department of Soil Science and Agricultural Chemistry, University of Agricultural Sciences, Bangalore, India

**Keywords:** Plant sciences, Environmental sciences

## Abstract

Silicate slags are one of the most widely used silicon (Si) source in agriculture. Even though the agronomic significance of slags has been demonstrated in several crops, only a few attempts were made to evaluate these Si sources based on their chemical composition. The main objective of this study was to characterize different silicate slags based on their chemical properties and to explore the effect of these chemical properties on the yield, and Si uptake in wetland rice, and dissolution of Si into the soil. Slags were characterised for pH, calcium and magnesium content (alkalinity, A), silicon content, 5 day Na_2_CO_3_ + NH_4_NO_3_ extractable Si content, and alkalinity to Si ratio (A/Si). Greenhouse and incubation experiments were also conducted using different silicate slags and wollastonite applied at the rate of 300 kg Si ha^−1^. Slags with A/Si < 3 were found to be ideal Si sources for the economic production of wetland rice and found consistent in increasing soil Si content and rice Si uptake. We conclude that the A/Si ratio of slags can be used as an important parameter to assess the agronomic efficiency of silicate slags in wetland rice.

## Introduction

Rice is the staple food crop of more than half of the world’s population. It is cultivated in about 160.9 m ha with a production of 719.7 mt under four major ecosystems viz. irrigated (57%), rainfed lowland (31%), rainfed upland (9%) and deep water (3%)^1^. Rice is considered as a typical Si accumulator plant and the beneficial effects of Si on stimulating plant growth and yield in rice have received increasing attention^2,3^.

The critical concentration of Si in rice straw was reported to be 2.9–3.4%^4,5^ and is capable of removing 470–1000 kg Si ha^−1^^6,7^. Soils of major rice growing regions, the tropics and sub-tropics are typically acidic and depleted of soluble sources of silicon (Si) due to weathering and leaching associated with high rainfall and temperatures, together with intensive cropping^8–12^. This emphasize the need for field application of Si source/sources, which can improve soil Si status and sustain better rice production.

Agronomic significance of many potential Si sources like calcium silicates, calcium magnesium silicate as metallurgic slags, potassium silicates, bagasse fly ash, diatomaceous earth, thermo-phosphates or fused magnesium phosphate, volcanic rock dust or crushed basalt, foliar/ liquid formulations, crop residues, biochar, etc*.* has been demonstrated in several studies^5,12–15^. Silicate slags is one of the widely used and a promising source of Si in agriculture^5,14,16^. Studies have shown that the total Si content of these sources were not a reliable indicator of how effective they release plant-available Si into soil nor facilitate its uptake by the plants^10,17^. There are only few attempts made to describe the agronomic performance of different Si sources and Si uptake based on their chemical composition^18,19^. Most of the studies were limited to extractants for plant available Si from these sources^12,20,21^. In this study, silicate slags were evaluated as Si sources based on their alkalinity and Si composition, extractable Si content, ability to release silicon, acquisition of released Si by plants and their performance on growth and yield of rice.

## Results and discussion

### Chemical properties of silicate slags used in the study

Silicate slags showed a wide variation in their chemical properties (Table 1). Wollastonite and all the silicate slags had an alkaline reaction with water at 1:100 ratio. CCE of different slags used in this study suggests that it has low to moderate liming ability^22–25^. Application of silicate slags were reported to be effective in correcting soil acidity and widely used as soil amendment^8,10,25,26^.

**Table 1:** Chemical properties of silicate slags used in the study.

Sl. no	Si sources	pH	CCE	CaO	Total Si	ESi	RESi	A/Si ratio
		1:100		(%)				
1	Wollastonite	9.32	78.51	55.75	15.41	3.98	25.83	1.69
2	Calcium silicate-1	9.87	75.61	43.29	11.45	2.38	20.79	1.77
3	Calcium silicate-2	8.89	74.89	43.35	10.82	1.64	15.16	1.87
4	Furnace slag	11.65	33.57	45.23	7.75	1.35	17.42	2.76
5	Caster slag-1	11.74	60.35	37.23	6.80	2.04	30.00	2.55
6	Caster slag-2	11.14	54.25	36.15	8.67	3.10	35.76	1.91
7	EAF slag	10.02	25.83	27.35	7.20	3.57	49.58	1.78
8	Steel mill slag-1	9.53	23.56	47.98	7.08	0.16	2.26	3.15
9	Steel mill slag-2	9.68	9.30	27.51	7.06	2.47	34.99	1.83
10	BOF LD slag-1	9.01	68.92	35.91	5.78	1.53	26.47	2.87
11	BOF LD slag-2	8.98	70.48	35.24	5.49	0.78	14.21	2.96
12	Desulfurization slag	8.59	4.37	27.35	3.76	0.67	17.82	3.38
13	Ladle slag-1	8.24	71.25	40.80	3.84	0.21	5.47	4.90
14	Ladle slag-2	10.44	77.48	52.43	4.06	0.49	12.07	6.03
15	Ladle slag-3	9.64	82.12	48.57	3.51	0.02	0.57	6.38

Alkalinity (CaO) of slags ranged from 27.35 to 52.43%. Total Si content ranged from 3.51 to 11.45%. A/Si ratio ranged from 1.83 to 6.38. The chemical composition observed in this study was in accordance with previous reporting where, silicate slags were reported to contain SiO_2_, CaO and A/Si ranging from 11.3–51.71%, 27.3–63.1% and 0.75–4.48 respectively^19,27^.

Extractable Si estimated by Na_2_CO_3_ + NH_4_NO_3_− 5 day incubation method (ESi) from the slags ranged from 0.02 to 3.57% and accounted only to 0.57 to 49.58% relative extractable Si (RESi) (Table 1). Highest Si content was reported in calcium silicate-1 (11.45%), whereas highest RESi was present in EAF slag (49.58%). Least Si content and RESi was noted in Ladle slag- 3. Wollastonite used in this study contain 15.4% of Si, of which 25.83% was RESi (Table 1). Wollastonite and silicate slags were reported to contain 2.2–3.6% ESi and 0.0–19% RESi^17,20^.

Total Si content of the slags seldom represents the bioavailable fraction of Si in them. A significant correlation was found between total Si content of slags and ESi, but, no correlation was observed between total Si content of slags with RESi (Table 2). RESi is the percent Si extracted by Na_2_CO_3_ + NH_4_NO_3_ method in relation to the total Si and this extractable part of Si was considered to be bioavailable to plants and found to correlate well with Si uptake in plants^17,20,21^.

**Table 2:** Correlation between different chemical properties of slags [*significant at *p* < 0.05, **significant at *p* < 0.01 (each data represents an average of three replication)].

Properties of Si sources	pH (1:100)	CaO (%)	Total Si (%)	ESi (%)	RESi (%)	A/Si ratio
pH(1:100)	1	ns	ns	ns	ns	ns
CaO (%)	0.099	1	ns	ns	*	ns
Total Si (%)	0.119	0.334	1	**	ns	**
ESi (%)	0.294	− 0.175	0.741	1	**	**
RESi (%)	0.349	− 0.576	0.328	0.854	1	**
A/Si ratio	− 0.142	0.366	− 0.716	− 0.773	− 0.686	1

A balanced ratio and amount of calcium, magnesium and silicon was considered to be one of the important characteristic of an ideal Si source, apart from other properties such as high soluble Si content and ready availability of this Si for plants^5,28^. However, such studies are limited and little attention has been made to study the effect of Si and Ca balance or A/ Si content of Si sources on bioavailability of Si. In the present study, ESi and RESi was found to have a strong significant negative correlation with A/Si of slags (*p* < *0.01*) (Table 2). That means, those slag materials with high alkalinity compared to total Si have less extractable or bioavailable Si. The potential of A/Si as an efficiency assessment factor for silicate slags were explored further by green house and incubation experiments.

## Greenhouse experiment

### Effect of slags on yield, Si content and Si uptake of wetland rice

There was a significant effect on straw and grain yield of rice with the application of Wollastonite and silicate slags (Table 3). Highest straw yield was recorded with the application of Caster slag-1(11.91 g/pot) which was on par with Wollastonite, Calcium silicate-1, Furnace slag, Caster slag- 2, EAF slag, steel mill slag-2, BOF LD slag-1 and BOF LD slag-2. Plants treated with furnace slag produced the highest grain yield and was significantly higher than Wollastonite and on par with other slags like Caster slags, EAF slag, BOF LD slags and calcium silicates. Slags such as caster slag and furnace slag could recorded relative yield higher than 70% which was significantly higher than that of Wollastonite (55.43%). Silicate slags are one of the widely used Si source and its beneficial effect to improve rice yield was reported from many countries like America^5,12,29^, Japan^30^, China^2,31^, Brazil^22,32^, South East Asian countries^33^ like South Korea, Thailand, the Philippine, Vietnam, Malaysia as well as in India^4,14,28,34–36^.

**Table 3:** Effect of slags on yield, Si content and Si uptake of wetland rice.

Treatments	Yield (g/pot)		Relative yield (%)	Si content (%)		Total Si uptake (g/pot)
	Straw yield	Grain yield		Straw	Grain	
Control	6.52	3.97	–	3.76	2.75	0.35
Wollastonite	11.23	5.06	55.43	6.60	4.38	0.96
Calcium silicate-1	10.40	5.44	50.94	7.06	4.31	0.97
Calcium silicate-2	9.94	5.36	45.76	6.59	4.16	0.88
Furnace slag	11.16	7.22	75.35	6.48	4.28	1.03
Caster slag-1	11.91	6.90	79.58	7.02	4.08	1.12
Caster slag-2	11.38	6.93	74.43	7.56	4.05	1.14
EAF slag	10.48	6.93	65.97	7.52	4.13	1.07
Steel mill slag-1	9.70	5.35	43.21	5.90	4.02	0.79
Steel mill slag-2	10.39	5.14	48.07	7.08	4.07	0.94
BOF LD slag-1	11.33	6.12	66.34	6.31	4.01	0.96
BOF LD slag-2	10.44	5.87	55.48	6.31	3.99	0.89
Desulfurization slag	10.02	4.53	38.41	5.30	4.00	0.71
Ladle slag-1	8.73	3.12	12.97	6.05	4.03	0.65
Ladle slag-2	6.68	4.59	7.38	6.33	3.94	0.60
Ladle slag-3	6.43	4.32	2.46	5.21	3.51	0.49
SEm ±	0.65	0.63	5.42	0.23	0.13	0.06
LSD (*p* < 0.05)	1.8	1.83	17.26	0.64	0.36	0.16

In many studies, Wollastonite was used for comparison to the other materials since a linear relationship was noticed between its rate of application and Si uptake by crops^17,22^. However, studies in which several slags performing better or on par with wollastonite is also not uncommon^22^. Significantly high straw and grain Si content was obtained with the application of different slags compared to control. Total Si uptake was significantly high in all slag treatments compared to control except ladle slag-3. Caster slags recorded the highest Si uptake (1.12–1.14 g/pot) which was on par with EAF slag and was significantly higher than that of Wollastonite (0.96 g/pot). Grain yield produced by desulfurization slag and ladle slags were on par with control. The relative yield produced by all the Ladle slags were significantly less than that of Wollastonite (Table 3). Bollich et al.^37^; Lee et al.^38^ and Yang et al.^25^ also demonstrated that the application of silicates to mineral soils does not consistently increase rice yield. Pereira et al.^27^ and Kato and Owa^19^ reported variation in agronomic performance of different silicate slags in rice and attributed this variation to dissolution of Si from these slags to soil and their uptake by the crop.

### Correlation between yield and Si uptake of rice and A/Si of slags

Linear correlations between yield and Si uptake of rice and different chemical properties of slags is shown in Table 4. ESi and RESi showed significant relationship with straw Si content and total Si uptake of rice (*p* < *0.01*). This is in accordance with previous findings^17,20^. A/Si of silicate slags also showed significantly higher correlation with straw yield, straw Si content, grain Si content and total Si uptake. Significant correlations between Si uptake of rice with RESi and A/Si ratio of slags proves that the extractable fraction of Si from slag is bioavailable and is influenced by the A/ Si ratio of slags. Further, RESi and A/Si ratio of slags were also found to have strong correlation with relative yield *(p* < *0.01).*

**Table 4:** Correlation between different properties of slags, yield and Si content of wetland rice [*significant at *p* < 0.05, **significant at *p* < 0.01 (each data represents an average of three replication)].

Properties of Si sources	pH (1:100)	CaO (%)	Total Si (%)	ESi (%)	RESi (%)	A/Si ratio
Straw yield	0.264	− 0.386	0.518	0.633*	0.626*	− 0.878**
Grain yield	0.712**	− 0.263	0.285	0.519	0.632*	− 0.598*
Straw Si content	0.495	− 0.267	0.505	0.802**	0.812**	− 0.692**
Grain Si content	0.153	0.037	0.765**	0.638*	0.433	− 0.768**
Total Si uptake	0.513	− 0.350	0.549*	0.773**	0.787**	− 0.869**
Relative yield	0.492	− 0.362	0.457	0.637*	0.684**	− 0.826**

**Figure 1:**
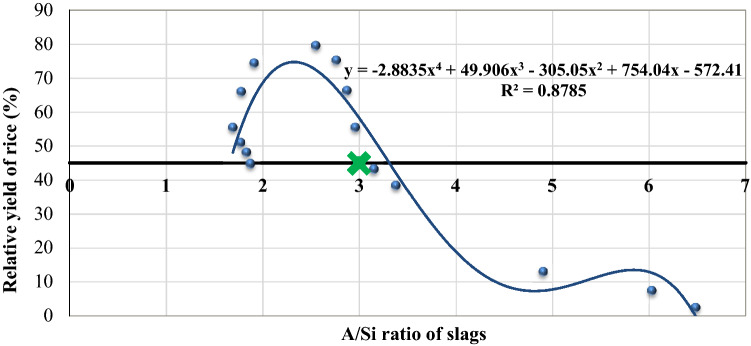
Relationship between A/Si of slags and relative yield of wet land rice [**significant at *p* < 0.01 (each data represents an average of three replication)].

Several regression analysis were made between different chemical properties of slag (Table 1) and plant parameters (Table 3) and a significant polynomial relationship was identified between A/Si of slags and relative yield of rice (r^2^ = 0.878, *p* < *0.01*). Addition of an agronomic management practice to be economical for the production of rice, rule of thumb is that, it should contribute to a 45% increase in yield^39^. It was observed that those slag sources with A/Si < 3 could produce relative yield more than 45% (Fig. 1).

## Soil incubation study

**Figure 2:**
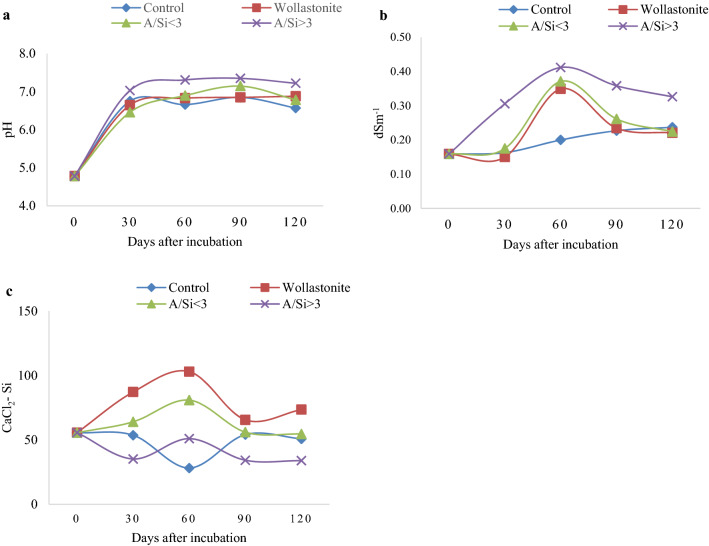
Change in pH (**a**), EC (**b**) and concentration of extractable Si (**c**) at different days after incubation in soil with the addition of different silicate sources. (each data represents an average of three replication; A/Si < 3-average of treatment with caster slag, furnace slag and EAF slag; A/Si > 3-average of treatment with laddle slag-2, laddle slag-3 and desulfurization slag replicated thrice).

An incubation experiment was conducted to study the effect of A/Si of slags on the dissolution of Si from these sources in the soil. Figure 2 depicts the trend in pH, EC and CaCl_2_ extractable Si (CaCl_2_-Si) in the soil at different intervals of incubation with wollastonite, slags with A/Si < 3(Caster slag, Furnace slag and EAF) and slags with A/Si > 3 (Laddle slag-2, ladle slag-3 and Desulfurization slag). The selection of silicate slags with A/Si < 3 and > 3 for this experiment was done based on the chemical characterization (Table 1) and green house experimental results (Table 3).

### Effect of slags on soil pH

A/Si > 3 slag treatments tend to increase the soil pH compared to other treatments, but there was no significant effect on soil pH with the application of Si sources over control. Irrespective of treatments, pH of the soil attained near neutral after 30 days of incubation (Fig. 2a). This could be due to three reasons: (1) submerged soil condition in which study was conducted: soil reaction was reported to attain fairly stable value of 6.7–7.2 a few weeks after submergence^40^, (2) CCE of the silicate slags used: A/Si < 3 slags have low CCE [EAF slag (25.83), Furnace slag (33.57) and Castor slag-2 (54.25)] and A/Si > 3 slags have moderate CCE [ladle slags (71.25 to 82.12)], and (3) Low rate of slag application: Here, silicate slags were used as Si source and rate of application was calculated to provide 300 kg Si ha^−1^. Accordingly, the lowest rate of application was for EAF slag (equivalent to 9 Mg of slag ha^−1^) and highest for ladle slag-3 (equivalent to 18.5 Mg slag ha^−1^). The liming requirement for the soil used in the study was calculated to be 25 Mg ha^−1^ of lime with 100% relative neutralizing value^41^.

### Effect of slags on soil EC

EC of the soil was significantly increased with the addition of wollastonite and slags (Fig. 2b). There was no significant difference in EC with the application of slags with A/Si < 3 and A/Si > 3. This can be due to the higher soluble salt content of these materials especially that of CaO and MgO, which react with water to form Ca(OH)_2_ and Mg(OH)_2_ and possess water solubility concentration of 1.20 g L^−1^ and 0.009 g L^−1^, respectively^42^_._

### Effect of slags on CaCl_2_ extractable Si in soil

Wollastonite, followed by slags with A/Si < 3 recorded significantly higher CaCl_2_–Si compared to control up to 90 DAI (Fig. 2c). However, in the present study, slags with A/Si > 3 were found to decrease CaCl_2_–Si in soil, which is contradictory to the earlier reports of Kato and Owa^19^ where slags with high A/Si ratio were reported to be ideal as Si source based on the release of Si in to soil. This decrease in CaCl_2_–Si with the application of slags with higher A/Si ratio can presumably due to the release of large amount of Ca and increase in soil pH, which were found to depress the continuous dissolution of the slags and enhance the specific adsorption of silicic acid by the soil^18^.

Correlation coefficient calculated between the CaCl_2_–Si released at different incubation interval and total Si uptake by rice were 0.5986, 0.9097**, 0.4381, and 0.5102 at 30. 60, 90 and 120 DAI respectively. The increase in silicon concentration at 60 DAI was found to be strongly correlated to Si uptake (r = 0.9097**, *p* < *0.01*). An increase in soil Si concentration due to dissolution of silicates at the early growth stages of paddy and increased uptake of Si with the application of slags was reported previously also^19^.

In general, slags with A/Si < 3 showed to release significantly higher amount of CaCl_2_–Si compared to control and slags with A/Si > 3 (Fig. 2). Even though, the CaCl_2_–Si released in soil by slags with A/Si < 3 (Caster slag, Furnace slag and EAF) were lesser than that of wollastonite, they found to be more bioavailable as evident with significant increase in Si content and Si uptake in plants treated with these slags (Table 3). Plant available Si estimated by Na_2_CO_3_ + NH_4_NO_3_− 5 day incubation method and relative extractability of Si from slags with A/Si < 3 were also found to be high. Slags with A/Si > 3 were found to decrease CaCl_2_–Si in soil, plant Si content, Si uptake and yield of rice. Hence, it is summarized that slags with A/Si < 3 such as caster slag-1, furnace slag, caster slag-2, EAF slag, calcium silicate-1, steel mill slag-2, BOF LD slag-1 and BOF LD slag-2 can be used as ideal Si source for the economic production of wetland rice.

## Conclusions

In this study, different silicate slags were characterized based on their chemical properties, their effect on yield, Si uptake of wetland rice, and dissolution of Si into the soil. This study could bring out the importance of having balanced ratios and amounts of calcium (Ca), magnesium (Mg), and silicon in an ideal Si source and found that A/Si of slags can influence the agronomic potential of silicate slags. Slags with A/Si < 3 like caster slag, furnace slag, EAF slag, slag based calcium silicate, steel mill slag and BOF LD slag were found to have higher Na_2_CO_3_ + NH_4_NO_3_ extractable Si content, could increase plant- available Si in soil, Si uptake, and can increase rice production. Slags with less A/Si < 3 can be used as potential Si sources in wetland rice.

## Materials and methods

### Chemical characterization of Si sources

The Si sources used in this experiment were Wollastonite (Sigma Aldrich, Merck) which was used as a standard Si source^22^, two slag based calcium silicates (Harsco *Pvt. Ltd.*) and twelve metallurgic slags (from various steel mills across India). Slags were dried till constant weight in a hot air oven, pulverised with a hardened steel pounder and sieved through 100 mesh screen (0.149 mm sieve opening) and further used for characterization, greenhouse and incubation study. Aqua regia extractable (EPA method 3050 B) nutrient content in the Si sources used ranged from 0–0.2% P, 0–2.6% Fe, 0–1.1% Mg and < 0.5% Zn, Cu and B.

#### CaO content (Alkalinity, A)

The sum of calcium and magnesium contents in the slags was extracted by boiling with 0.5 M HCI for 5 min and expressed as CaO (%)^19^.

#### Calcium carbonate equivalent (CCE)

CCE was analysed using AOAC methodology^23^. In this study, the slag was ground to pass a 100 mesh sieve before application, thus the relative neutralizing value (RNV) was considered as 100%^24^.

#### Total Si content

Silicon content in digested samples were estimated colorimetrically. Microwave digestion technique was adopted for digesting slag samples. 25 mg source material was pre- digested in a mixture of 7 ml of HNO_3_ (70%), 2 ml of H_2_O_2_ (30 per cent) and 1 ml of HF (40%) for 15 min in Teflon vessels, digestion was done using microwave digestion system (Milestone-start D) in SK-10 T high pressure segmented rotor with following programme: 1000 watts for 25 min temperature set at 200 °C with a ramping rate of 7 °C per minute; 1000 watts for 15 min at holding temperature of 200 °C; venting for 10 min. Volume of the digested matrix was made up to 50 ml with 4% boric acid immediately. Extracted Si was estimated as described below^43^. An aliquot of 0.25 ml digested extract was taken into a plastic centrifuge tube and in to it 10.5 ml of distilled water, 0.25 ml of 1:1 hydrochloric acid, and 0.5 ml of 10 per cent ammonium molybdate solution were added. After allowing for 5 min, 0.5 ml of 20 per cent tartaric acid solution was added. After allowing for additional two minutes, 0.5 ml reducing agent (1-amino-2-napthol-4-sulfonic acid-ANSA) was added. After 5 min, but not later than 30 min following addition of the reducing agent, absorbance was measured at 630 nm using UV–visible spectrophotometer (SHIMADZU Pharmaspec, UV-1700 series). Si standards (0, 0.2, 0.4, 0.8, 1.2, 1.6, 1.8 and 2 mg L^−1^) prepared in the same matrix were also measured to set the standard curve.

#### Alkalinity- silicon ratio (A/Si)

It is the ratio of alkalinity (A) to total Si content of slag^19^.

#### 5-Day Na_2_CO_3_ + NH_4_NO_3_ extractable Si (ESi)

Extractable Si was estimated by Na_2_CO_3_ + NH_4_ NO_3_ extraction^17,20^. This 5-Day Na_2_CO_3_ + NH_4_NO_3_ soluble Si extraction method has been accepted as the official method in the United States for determining plant-available Si from non- liquid fertilizers^20^.

#### Relative percentage of extractable Si (RESi)

Relative percentage of extractable Si is calculated by comparing percentage Si extracted by the extractant to total Si extracted by microwave digestion. The relative percentage of Si^17,20^ extracted was calculated using the following formula:

$$Relative\;extractable\;{\text{Si }}\left( \% \right) \, = \, \left( {Si\;extracted\;by\;extractant\;*\;100} \right)/\left( {total\;{\text{ Si}}\; \, content} \right)$$

### Greenhouse experiment

Pot culture experiment was conducted at the greenhouse of Department of Soil Science and Agricultural Chemistry, University of Agriculture sciences, Bangalore, India. Bulk soil sample for the experiment were collected at a depth of 10 to 20 cm from Hassan representing southern dry zone of Karnataka, South India with characteristic properties of *Rhodic Paleustalfs* with acidic pH of 4.78. The soil was air dried and sieved through a 2 mm screen and then its initial chemical and physical properties were determined^44^ (Table 5). Plant available Si in soil was estimated by 0.01 M CaCl_2_ extraction^36,45^.

**Table 5:** Initial properties of soil used in this experiment.

Soil parameters	
Location	Hassan, Karnataka
**Particle size distribution**	
Sand (%)	51.47
Silt (%)	20.37
Clay (%)	26.17
Soil texture	Sandy clay loam
pH (1:2.5 soil: water ratio)	4.78
EC (dS m^−1^)	0.16
OC (g kg^−1^)	8.3
Available N (kg ha^−1^)	466.46
Available P_2_O_5_ (kg ha^−1^)	163.69
Available K_2_O (kg ha^−1^)	495.14
Available S (mg kg^−1^)	13.1
Exchangeable Ca (cmol (*p*^+^) kg^−1^)	6.01
Exchangeable Mg (cmol (*p*^+^) kg^−1^)	3.2
Exchangeable Na (cmol (*p*^+^) kg^−1^)	0.18
CaCl_2_ –Si (mg kg^-1^)	56.42

Rice plants were grown in 5 kg soil taken in closed plastic pots (20 cm upper diameter × 17 cm height) maintained under submergence. Fifteen Si sources were applied at the rate of 300 kg Si ha^−1^ and a control without any Si application. The experiment was set up in completely randomized block design with three replications. One seedling of 21 day old rice (*var.* BR 2655) was transplanted to each pot. Fertilizers were mixed with soil at the recommended dose of 100:50:50 kg ha^−1^ N:P_2_O_5_:K_2_O as urea, single super phosphate and potassium chloride, respectively. The straw and grain samples were collected at harvest, oven dried at 60 °C and recorded the dry weight. Later, the samples were powdered in a Retsch miller mill MM400 with Tungsten balls and analysed for Si content^43^. Total Si uptake (straw plus grain Si uptake) and relative yield for each treatment was calculated using following formulae.

$$\begin{aligned} Total\;{\text{Si}}\;uptake\left( {g/pot} \right) & = \left( {Straw\;yield\; \times \;straw\;{\text{Si}}\;content/100} \right) \\ & \quad + (grain\;yield\; \times \;grain\;{\text{Si}}\;content/100) \\ \end{aligned}$$

$$Relative\;yield \, \left( \% \right) \, = \, [\left( {Yield\;in\;treatment\; - \;Yield\;in\;control} \right)\; \times \;100]/yield\; \, in\; \, control$$

### Incubation experiment

An incubation study was also conducted with selected six slags viz*.* caster slag, furnace slag, EAF slag, laddle slag-2, laddle slag-3 and desulfurization slag in addition to wollastonite, to investigate the dissolution and release of silicon from these sources. 200 g soil was mixed with calculated amount of sources to provide 300 kg Si ha^−1^ and kept under submergence in closed plastic pots (10 cm upper diameter × 8.5 cm height). Twelve replications of each treatment and control (soil with no added Si sources) were kept and sampling was done at four intervals: 30, 60, 90 and 120 DAI (days after incubation). At each interval, destructive sampling of three replications were done. Soil pH, EC and 0.01 M CaCl_2_ extractable Si^36,45^ were recorded at each interval.

### Statistical analysis

Data generated were statistically analysed using one-way ANOVA by using Fisher's test at *p* ≤ 0.05 and correlation–regression analysis were done using XLSTAT software.
